# Exploration of the Diversity of Clustered Regularly Interspaced Short Palindromic Repeats-Cas Systems in *Clostridium novyi sensu lato*

**DOI:** 10.3389/fmicb.2021.711413

**Published:** 2021-09-13

**Authors:** Thibault Le Gratiet, Caroline Le Marechal, Marie Devaere, Marianne Chemaly, Cédric Woudstra

**Affiliations:** ^1^Hygiene and Quality of Poultry and Pig Products Unit, ANSES, French Agency for Food, Environmental and Occupational Health Safety, Ploufragan, France; ^2^UFR of Life Sciences and Environment, University of Rennes, Rennes, France; ^3^Department of Veterinary and Animal Sciences, University of Copenhagen, Frederiksberg, Denmark

**Keywords:** CRISPR-Cas, type V-U CRISPR, Cas14a, phage-inducible chromosomal island, protospacers, mobilome, *Clostridium botulinum* group III

## Abstract

Classified as the genospecies *Clostridium novyi sensu* lato and distributed into four lineages (I–IV), *Clostridium botulinum* (group III), *Clostridium novyi*, and *Clostridium haemolyticum* are clostridial pathogens that cause animal diseases. *Clostridium novyi sensu lato* contains a large mobilome consisting of plasmids and circular bacteriophages. Here, we explored clustered regularly interspaced short palindromic repeats (CRISPR) arrays and their associated proteins (Cas) to shed light on the link between evolution of CRISPR-Cas systems and the plasmid and phage composition in a study of 58 *Clostridium novyi sensu lato* genomes. In 55 of these genomes, types I-B (complete or partial), I-D, II-C, III-B, III-D, or V-U CRISPR-Cas systems were detected in chromosomes as well as in mobile genetic elements (MGEs). Type I-B predominated (67.2%) and was the only CRISPR type detected in the Ia, III, and IV genomic lineages. Putative type V-U CRISPR Cas14a genes were detected in two different cases: next to partial type-IB CRISPR loci on the phage encoding the botulinum neurotoxin (BoNT) in lineage Ia and in 12 lineage II genomes, as part of a putative integrative element related to a phage-inducible chromosomal island (PICI). In the putative PICI, Cas14a was associated with CRISPR arrays and restriction modification (RM) systems as part of an accessory locus. This is the first time a PICI containing such locus has been detected in *C. botulinum*. Mobilome composition and dynamics were also investigated based on the contents of the CRISPR arrays and the study of spacers. A large proportion of identified protospacers (20.2%) originated from *Clostridium novyi sensu lato* (p1_Cst, p4_BKT015925, p6_Cst, CWou-2020a, p1_BKT015925, and p2_BKT015925), confirming active exchanges within this genospecies and the key importance of specific MGEs in *Clostridium novyi sensu lato*.

## Introduction

*Clostridium novyi sensu lato* is a genospecies, containing the closely related species *Clostridium botulinum* (group III), *Clostridium novyi*, and *Clostridium haemolyticum* (Skarin et al., 2011; Skarin and Segerman, 2014). This proposed genospecies (Skarin et al., 2011) is based on genomic comparisons: their chromosome is highly conserved and the differences between these three species can be attributed to the content of their mobile genetic elements (MGEs; Skarin and Segerman, 2011). These three clostridia are ubiquitous, anaerobic, Gram-positive, and spore-forming pathogens that can affect both humans and animals. Botulinum neurotoxins (BoNTs) produced by *C. botulinum* group III are responsible for animal botulism. Alpha, beta, and gamma toxins produced by *Clostridium novyi* cause gas gangrene in humans and animals and black disease in animals, mostly in sheep. The beta toxin produced by *C. haemolyticum* is responsible for bacillary hemoglobinuria affecting ruminants (Skarin and Segerman, 2014). These virulence genes are all carried on MGEs (plasmids and phages) as part of their mobilome. The *Clostridium novyi sensu lato* mobilome is highly diverse: More than 60 plasmids or circular bacteriophages (called phages hereafter) categorized into 13 groups were detected upon sequencing only 24 genomes (Skarin and Segerman, 2014). Considering the few genome sequences available in the public databases, exploring further *Clostridium novyi sensu lato* MGE diversity is difficult. Yet, clustered regularly interspaced short palindromic repeats (CRISPR) and CRISPR-associated proteins (Cas), together making up the CRISPR-Cas system, which records MGEs past encounters, could help to shed light on a putative larger mobilome within *Clostridium novyi sensu lato*.

Found in bacteria and archaea, CRISPR–Cas is a prokaryotic adaptive immunity system that interferes with invading phages and plasmids (Koonin and Makarova, 2019). CRISPR systems are composed of CRISPR arrays, divided into repeats and spacers (short variable DNA sequences), and *cas*-associated genes. Spacers, which are of foreign origin (protospacers), are integrated into the CRISPR locus, thereby acting as a memory of previous infections. Analysis of these spacers can give clues as to the mobile elements encountered in the past by a given organism (Mcginn and Marraffini, 2019), thus providing information on the identity of horizontally transferable elements and their interaction with their bacterial hosts in their environment. The immune function of CRISPR is carried out by Cas proteins in three successive steps (adaptation, expression, and interference; Briner et al., 2015; Makarova et al., 2020; Pan et al., 2020). Briefly, during the adaptation stage, a part of the foreign DNA (i.e., protospacers) is incorporated into CRISPR arrays. Then, these CRISPR arrays are transcribed to generate short processed CRISPR RNA (crRNA). The last stage consists of crRNA guiding Cas proteins to their DNA target and finally cleavage of the target. With the ever-increasing number of available bacterial genomes, the classification of CRISPR-Cas systems is constantly evolving (Makarova et al., 2020). Currently, two classes, six types, and more than 20 subtypes of CRISPR-Cas systems have been described based on *cas* gene content (Mcginn and Marraffini, 2019; Makarova et al., 2020).

Up to now, only one study has explored *in silico C. botulinum* CRISPR-Cas systems, with an analysis of 20 genomes (mainly draft sequences) demonstrating 80% prevalence of CRISPR loci in *C. botulinum* (Negahdaripour et al., 2017). Most of the studied strains contained several CRISPR loci, with one-fifth located on plasmids, although CRISPR loci are commonly found on circular chromosomes in bacteria (Negahdaripour et al., 2017). Once again, this particularity attests to the importance of the *C. botulinum* mobilome. Only eight strains of *C. botulinum* group III (including seven BoNT type C/D and one type D, from genomic lineages Ia, Ib, III, and IV) were included in this study and led to the detection of CRISPR loci and CRISPR arrays, demonstrating the existence of CRISPR systems in *C. botulinum* group III strains (Negahdaripour et al., 2017). However, with only a few genome sequences analyzed, the full diversity of the CRISPR content of *C. botulinum* group III has not been fully explored. Further investigations are therefore required to better characterize the CRISPR-Cas systems and their spacers memory in *C. botulinum* group III genomes and more generally in the *Clostridium novyi sensu lato* genospecies. The objective of our study was thus to explore the CRISPR-Cas systems in the *Clostridium novyi sensu lato* genospecies to evaluate their presence, determine their characteristics, and explore the protospacer origins to gain insight into the MGEs interacting in this taxon.

## Materials and Methods

### Genome Selection

Fifty-eight genomes (Table 1) available in the GenBank database (National Center for Biotechnology Information, NCBI; https://www.ncbi.nlm.nih.gov/) as of January 2021 were retrieved to explore the CRISPR-Cas systems in the *Clostridium novyi sensu lato* genospecies. Seven of them were complete genomes (BKT015925, C-Stockholm, BKT2873, 1873, 3859/11, 150557, and NT) and the remaining 51 were draft genomes: 45 belong to *C. botulinum* (BoNT/C, C/D, /D, and D/C), 10 belong to *Clostridium novyi*, and three belong to *C. haemolyticum*, showing high variability in sampling year, location, country, sample origin, and genomic lineage. The *Clostridium novyi sensu lato* genospecies, defined according to a previously published classification (Skarin and Segerman, 2011), fall into four different lineages, based on genomic comparisons using Gegenees (Agren et al., 2012), a software tool using a fragmented alignment approach to compare bacterial genomes (Skarin and Segerman, 2014).

**Table 1:** Strain metadata and clustered regularly interspaced short palindromic repeats (CRISPR)-CRISPR-associated proteins types.

Strain	Species	Estimated genome Size (Mbp)	Location	Year	Origin	BoNT type	Lineage^a^	GenBank access number	CRISPR type	CRISPR location	Number of spacers in the genome
Eklund	*Clostridium botulinum*	2.96	United States	/	/	C	III	ABDQ00000000.1	I-B	chr	20
K25	*Clostridium* sp.	2.60	South-Korea	2012	Pig	NT	II	JENU00000000.1	I-B, V-U	/	91
Stockholm	*Clostridium botulinum*	2.70	Sweden	1946	Mink	C	II	CP063816-CP063821	I-B, V-U	chr, p1, pCLG2/pCN2	85
IFR 18/084	*Clostridium botulinum*	2.61	/	/	/	C	II	SXEF00000000	I-B, V-U	chr	118
Colworth BL165	*Clostridium botulinum*	2.49	United Kingdom	1970	/	C	II	SWNS00000000	I-B, V-U	chr	135
571C	*Clostridium botulinum*	2.62	United States	/	/	C	II	SWUK00000000	I-B, V-U	chr	121
IFR 18/049	*Clostridium botulinum*	2.41	/	/	/	(C)	II	SWNT00000000	I-B, V-U	chr	91
7221C	*Clostridium botulinum*	2.68	/	/	/	C	II	SXDK00000000	I-B, V-U	chr	133
Davies AO	*Clostridium botulinum*	2.56	/	/	/	C	II	SXEV00000000	I-B, V-U	chr	119
IFR 18/078	*Clostridium botulinum*	2.46	/	/	/	(C)	II	SXES00000000	I-B, V-U	chr	51
12LNR10	*Clostridium botulinum*	3.04	France	2012	Turkey	C/D	Ia	LGVQ00000000	I-B	p1, p2	83
12LNR13	*Clostridium botulinum*	3.07	France	2012	Chicken	C/D	Ia	LGVT00000000	I-B	chr, p1	81
12LNRI	*Clostridium botulinum*	3.00	France	2012	Duck	C/D	Ia	LGVR00000000	I-B	p1, p2	73
29401	*Clostridium botulinum*	3.04	France	2008	Chicken	C/D	Ia	LGVP00000000	I-B	p1, p2	76
38028	*Clostridium botulinum*	3.12	France	2008	Chicken	C/D	Ia	LGVO00000000	I-B	p1, p2	75
43243	*Clostridium botulinum*	3.00	France	2009	Guinea Fowl	C/D	Ia	LGVU00000000	I-B	p1, p2	72
48212	*Clostridium botulinum*	3.00	France	2008	Duck	C/D	Ia	LGVS00000000	I-B	p1, p2	90
49511	*Clostridium botulinum*	3.09	France	2008	Chicken	C/D	Ia	LHYP00000000	I-B	chr, p1, p2	65
50867	*Clostridium botulinum*	3.08	France	2008	Chicken	C/D	Ia	LHYQ01000025	I-B	p1, p2	84
55741	*Clostridium botulinum*	3.04	France	2008	Turkey	C/D	Ia	LHYR00000000	I-B	p1, p2	83
58272	*Clostridium botulinum*	3.07	France	2008	Chicken	C/D	Ia	LHYS00000000	I-B	p1, p2	87
58752	*Clostridium botulinum*	2.82	France	2008	Chicken	(C/D)	Ia	LHYT00000000	I-B	p2	45
69285	*Clostridium botulinum*	2.94	France	2008	Chicken	(C/D)	Ia	LHYU00000000	I-B	p2	45
71840	*Clostridium botulinum*	3.00	France	2008	Chicken	C/D	Ia	LHYV00000000	I-B	p1, p2	84
BKT2873	*Clostridium botulinum*	3.19	Sweden	2007	Chicken	C/D	Ib	CP063965-CP063968	I-B	chr, p1	80
BKT12695	*Clostridium botulinum*	2.75	Sweden	2010	Chicken	(C/D)	III	JENP00000000	I-B	chr	68
BKT015925	*Clostridium botulinum*	3.20	Sweden	2008	Chicken	C/D	Ia	CP002410.1	I-B	p1, p2	83
BKT028387	*Clostridium botulinum*	2.83	Sweden	2007	Chicken	(C/D)	Ia	AESB00000000	I-B	p2	45
BKT75002	*Clostridium botulinum*	3.14	Denmark	2010	Chicken	C/D	Ib	JENS00000000	I-B	chr, p1	80
It1	*Clostridium botulinum*	2.50	Italy	/	Bovine	(D/C?)	IV	JENO00000000	/	/	0
Sp77	*Clostridium botulinum*	3.06	Spain	2011	Duck	C/D	Ia	JENQ00000000	I-B	p1, p2	77
V891	*Clostridium botulinum*	3.17	Sweden	2007	Gull	C/D	Ia	AESC00000000	I-B	chr, p1	71
1873	*Clostridium botulinum*	2.57	Chad	1958	Ham	D	II	CP063822-CP063828	I-B	chr, p1, pCLG2/ pCN2	22
16868	*Clostridium botulinum*	3.08	Netherlands	2001	Bovine	D	Ia	JENR00000000	I-B	p1, p2	91
CCUG7971	*Clostridium botulinum*	2.81	South Africa	1926	/	(D)	Ib	JDRZ00000000	I-D, II-C	/	26
3859/11	*Clostridium botulinum*	2.89	Italy	2011	Bovine	D/C	Ib	CP063959-CP063964	I-B, I-D, III-B	chr, p2	69
1274	*Clostridium botulinum*	2.94	Brazil		Vaccine strain	D/C	Ib	MVIY00000000	I-D, II-C	/	70
1275	*Clostridium botulinum*	2.94	Brazil	/	Vaccine strain	D/C	Ib	MVIZ00000000	I-D, II-C	/	70
1276	*Clostridium botulinum*	2.91	Brazil	/	Vaccine strain	D/C	Ib	MVJA00000000	I-D, II-C	/	70
1277	*Clostridium botulinum*	2.57	Brazil	/	Vaccine strain	D/C	Ib	MVJB00000000	II-C	/	32
47295	*Clostridium botulinum*	3.18	France	2009	Bovine	D/C	Ib	LHYX00000000	I-B, I-D, III-B	/	43
51714	*Clostridium botulinum*	3.18	France	2009	Bovine	D/C	Ib	LHYY00000000	I-B, I-D, III-B	/	48
CP05	*Clostridium botulinum*	2.88	Brazil	/	Vaccine strain	D/C	Ib	MVJC00000000	I-D, II-C	/	46
DC5	*Clostridium botulinum*	3.32	Italy	/	Bovine	D/C	Ib	JDRY00000000	I-B, I-D, III-B	/	85
LNC5	*Clostridium botulinum*	2.89	New Caledonia	2013	Bovine	D/C	Ib	LHYW00000000	I-D, II-C	/	39
4540	*Clostridium novyi*	2.50	Great Britain	2000	Human	NT	IV	JENL00000000	/	/	0
4552	*Clostridium novyi*	2.80	Great Britain	2000	/	NT	III	JENJ00000000	I-B	/	126
4,570	*Clostridium novyi*	2.32	Great Britain	2001	/	NT	IV	JDRX00000000	I-B	/	200
BKT29909	*Clostridium novyi*	2.46	Sweden	2007	Chicken	NT	IV	JENM00000000	/	/	4
GD211209	*Clostridium novyi*	2.46	Netherlands	2009	Bovine	NT	IV	JENN00000000	/	chr	25
NCTC538	*Clostridium novyi*	2.52	Great Britain	1920	Human	NT	IV	JENK00000000	I-B	/	94
ATCC27606	*Clostridium novyi*	2.61	Germany	/	/	NT	II	JENW00000000	I-B, I-D, III-B	/	112
NCTC9691	*Clostridium novyi*	2.64	Great Britain	1955	Sheep	NT	II	JENV00000000	I-B, I-D, V-U	/	77
150557	*Clostridium novyi*	2.30	South Korea	2015	Pig	NT	II	NZ_CP029458	I-B, III-D, V-U	chr, pCLG2 /pCN2	78
NT	*Clostridium novyi*	2.55	/	/	/	NT	IV	NC_008593.1	I-B	chr	96
NCTC9693	*Clostridium haemolyticum*	2.61	United States	1955	/	NT	II	JENX00000000	I-B	/	39
KFSHRC_CH1	*Clostridium haemolyticum*	2.87	Saudi Arabia	2014	Human	NT	II	NZ_LSZB01000000	I-B, V-U	/	209
NCTC8350	*Clostridium haemolyticum*	2.47	United States	1946	/	NT	II	NZ_JDSA00000000	I-B	/	38

A slash indicates that no information could be retrieved. For the *Clostridium botulinum* strains, the botulinum neurotoxin (BoNT) type is indicated in parentheses when the BoNT phage has been lost and is not present in the genome sequences. The *Clostridium novyi* and *Clostridium haemolyticum* strains are non-toxigenic (NT). Complete genomes are indicated in gray, all others are draft sequences.

a According to Skarin et al. (2011).

(), incomplete or lost BoNT phage p1; /, no information retrieved; NT, non-BoNT-producing strain.

### CRISPR-Cas Systems Identification

The CRISPRCasFinder server (https://crisprcas.i2bc.paris-saclay.fr/, University of Paris-Saclay, France) was used with default parameters (Couvin et al., 2018) to identify CRISPR loci, as well as to determine the presence and content of *cas* genes. The genetic environment of solitary CRISPR arrays with no predicted CRISPR locus was further explored using the HHpred server (https://toolkit.tuebingen.mpg.de/tools/hhpred/, Max Planck Institute, Germany; Zimmermann et al., 2018) with default parameters to search for undetected *cas* genes. Most of the available genomes were draft sequences, which may have resulted in incomplete or partial CRISPR-Cas loci identification.

### *In silico* Analysis of the Spacer Content in CRISPR Arrays

Clustered regularly interspaced short palindromic repeats arrays were identified using the CRISPR Recognition tool (CRT), software designed to detect CRIPSR arrays (Bland et al., 2007). Settings were chosen so as to have at least three repeats in the CRISPR array with a repeat length between 19 and 38 base pairs (bp) and a spacer length between 19 and 48bp. The spacer content of each CRISPR array was then extracted manually. Identical spacers were detected in several genomes and were removed to keep only one copy of each spacer that will be called “unique spacer” in the rest of the study. Complementary spacers in the reverse strand or harboring one or two mismatches were considered as unique spacers. Unique spacers were then screened with a Basic Local Alignment Search Tool (BLAST) search to identify protospacers using the CRISPRTarget tool (http://crispr.otago.ac.nz/CRISPRTarget/crispr_analysis.html, University of Otago, New Zealand; Biswas et al., 2013). Only matches in the GenBank-Phage and Refseq-plasmid databases with at least 80% homology were considered. In addition, only the available complete (not draft) genomes were used in these databases. Parameters for the initial BLAST screen were gap open=−10 and extend=−2, minimum BLAST score=21 with nucleotide match=1 and mismatch=−2, E value=1, and Word=7. Species targeted, target name and type, location in the genome, and gene function (for the main MGEs detected) were reported. Protospacer-adjacent motif (PAM) sequences associated with identified protospacers were reported when identified. In these databases, some MGEs had different names although they were identical (e.g., pCN2 is identical to pCLG2). Unnamed plasmids were designated with a “p_” followed by their strain name and number.

### Anti-CRISPR Genes Search

Anti-CRISPR proteins were searched on *Clostridium novyi sensu lato* plasmidome using AcRanker,^1^ a machine learning system for direct identification of anti-CRISPR proteins (Eitzinger et al., 2020).

## Results

### Overview of CRISPR-Cas Types Detected in *Clostridium novyi sensu lato*

**Figure 1:**
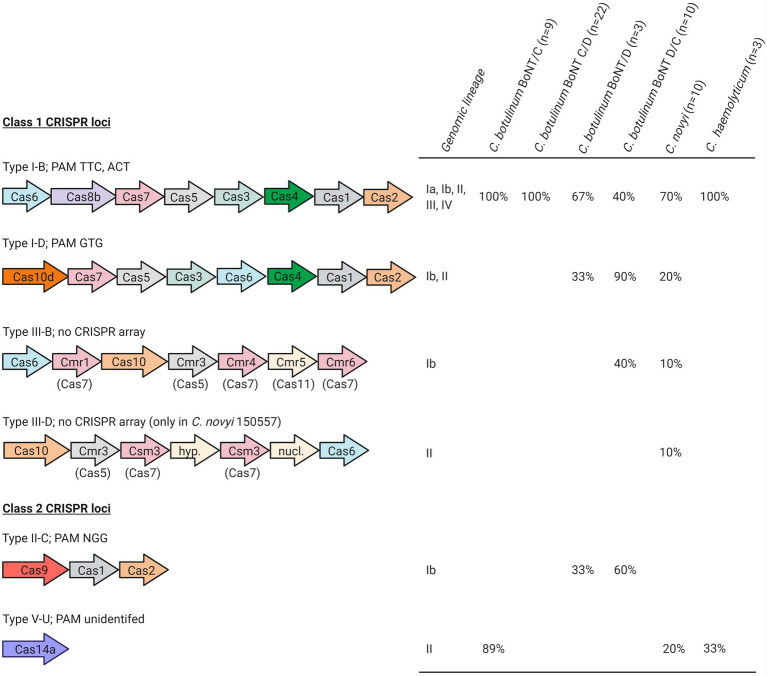
*Clostridium novyi sensu lato* CRISPR types. CRISPR types were identified throughout *Clostridium novyi sensu lato* genomes using the CRISPRCas++ server (Couvin et al., 2018). *Cas* genes are shown according to their original order in the genome sequences, in accordance with the literature (Koonin et al., 2017). Created with BioRender.com.

Six different CRISPR-Cas system types belonging to class 1 and 2 CRISPR loci were identified in 55 out of the 58 *Clostridium novyi sensu lato* strains included in our study, located either on the chromosome or on MGEs (BoNT phages, plasmids p2BKT015925, pCLG2/pCN2; Table 1; Figure 1). Twenty to 209 spacers were detected per strain. *In silico*-identified PAM sequences associated with type I-B, I-D, and II-C CRISPRs matched with canonical PAMs (Kieper et al., 2018; Figure 1). Type I-B CRISPR predominated and was detected in all lineages (Figure 1). Type I-D was found in 10 *C. botulinum* BoNT/D and D/C strains and in two *Clostridium novyi* strains (lineages I-b and II). Type III-B was detected in four *C. botulinum* BoNT D/C and one *Clostridium novyi* strains (lineage I-b; Table 1). The type III-B CRISPR-Cas locus appeared to be devoid of the Cas proteins involved in spacer acquisition (Cas1 and Cas2) and located in close vicinity to the type I-B CRISPR locus (among the complete available genomes). *Clostridium novyi* strain 150557 (lineage II) seems to possess a type III-D CRISPR locus. Among class 2 CRISPR loci, canonical type II-C was present in seven *C. botulinum* BoNT/D and D/C strains (lineage Ib) and type V-U in 12 *C. botulinum* BoNT/C, *Clostridium novyi*, and *C. haemolyticum* strains (lineage II).

**Figure 2:**
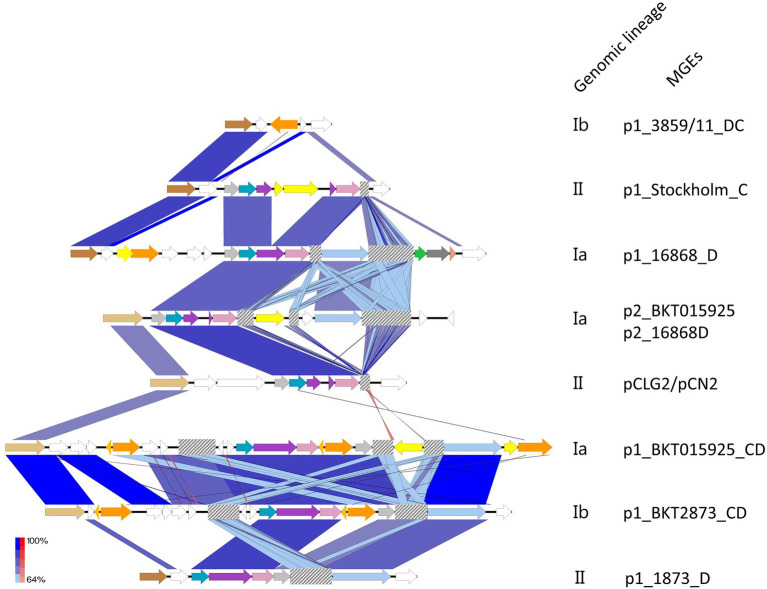
Comparison of the architecture of the CRISPR loci located in MGEs. Annotated CDS are shown in white. CRISPR loci were always found in close proximity to a replication initiation protein (first CDS on the left, dark and light brown). TnpB Cas14a related transposases are shown in orange; hypothetical transposase regulators, light orange; the IS256-like element ISCbo4 family transposase integrated into the CRISPR arrays of p1_BKT015925 and p2_BKT015925, yellow. CRISPR-Cas type Ib, arrows color coded as in Figure 1 (light gray=Cas5, Blue indigo=Cas6, Purple=Cas8b, Pink=Cas7, Light blue=Cas3, Green=Cas4, Dark grey=Cas1, and Orange=Cas2). CRISPR arrays are shown as gray-checkered boxes. BLAST hits are colored on a gradient ranging from minimum (light blue) to maximum BLAST scores (dark blue). Reverse matches are shown in a red gradient. Created with Easyfig (Sullivan et al., 2011).

Clustered regularly interspaced short palindromic repeats-Cas genes were found either in the chromosome or in MGEs (in which case, they were located on BoNT phages and plasmids, as illustrated in Figure 2). When located on MGEs, the CRISPR loci were of type I-B. Some strains harbored up to three different type I-B CRISPRs (located in the chromosome, BoNT phages, and plasmids). They varied among MGEs. For example, the type I-B CRISPR locus from p1_BKT015925 was different from that of p2_BKT015925, whereas none were detected for p1_Eklund phage and p1_3859/11 phage carried instead a bacteriophage exclusion system (BREX; Goldfarb et al., 2015). Of the available p1 BoNT phage and plasmid sequences, two type I-B CRISPR-Cas loci with major differences were identified (Figure 2): one shared by p1_Stockholm, p1_16868 BoNT phages, and the p2_BKT015925 and pCLG2/pCN2 plasmids and a second one shared by the p1_BKT015925 and p1_BKT2873 and p1_1873 BoNT phages. Therefore, two different CRISPR-Cas loci were acquired independently in these MGEs. CRISPR loci were also shared between plasmids and phages: we detected a related CRISPR locus in the p1_16868 phage and p2_BKT015925 plasmid (Figure 2). Furthermore, the CRISPR loci were unrelated to chromosomal type I-B CRISPR loci (data not shown) and exhibited no link with the bacterial genomic lineages.

These CRISPR loci located on MGEs were mostly incomplete, except in the p1_16868 BoNT phage, which contain an entire type I-B CRISPR (effector and adaptation module; Koonin et al., 2017). Incomplete CRISPR loci had lost Cas4, Cas1, and Cas2, which constitute the adaptation module (in MGEs p1_BKT015925, p2_BKT015925, p1_BKT2873, and p1_1873), as well as the Cas3 endonuclease that cleaves the DNA target in the effector module (in the p1_Stockholm and pCLG2/pCN2 MGEs). Noteworthily, transposases interrupting the CRISPR loci were detected in p1_BKT015925, p1_BKT2873, p1_1873, and p1_Stockholm. In p1_BKT015925, up to five transposases were detected in close vicinity to CRISPR genes. Additionally, they showed similarities with TnpB and Cas14a [one of 376 amino acids (a.a.) with a 100% HHPRED probability of being Cas14a, E-value 3.6e-43, score 327.87; another of 470 a.a. with a Cas14a probability of 100%, E-value 2.9e-40, and score 342.39]. Detection of TnpB transposases next to Cas proteins (in BKT015925, BKT2873, 1873, and C-Stockholm located on the *bont* phage) raises the question of their potential role in the CRISPR-Cas system and possible connection with the evolution of their CRISPR loci.

### A Putative Phage-Inducible Chromosomal Island Found in *Clostridium novyi sensu lato*

**Figure 3:**
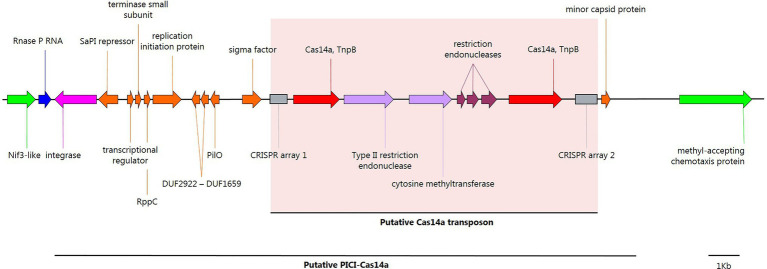
Annotated map of genes in a putative genetic phage-inducible chromosomal island (PICI; C-Stockholm strain) from *Clostridium novyi sensu lato* lineage II. Nif3-like and the methyl-accepting chemotaxis proteins surrounding the PICI site of insertion in the *C. botulinum* C-Stockholm (NZ_CP063816.1) strain are shown in bright green; integration targeted RNase P ncRNA, recognized by the PICI integrase, blue; PICI tyrosine-recombinase integrase, pink; PICI-related CDS, orange; type V-U CRISPR Cas14a, red; CRISPR arrays, gray boxes; and restriction-modification related CDS, light purple, and dark red. This figure was created with SnapGene.

Two other putative TnpB transposases (TnpB_IS605) were detected on the chromosome of *Clostridium novyi sensu lato* genomic lineage II, next to solitary CRISPR arrays (Table 1; Figure 3). HHPRED predicted that the TnpB transposases were homologous to class 2 type V-U CRISPR Cas14a with a probability of 100%, E-value 3.1e-38–1.6e-46, and score 327.01–367.87 (present in 12 strains of *C. botulinum* BoNT/C, *Clostridium novyi*, *C. haemolyticum*, and *Clostridium* sp.; Table 1). The two putative chromosomal *cas14a* genes encode homologous proteins (35.6% homology at the C terminus between the two genes) conserved among 11 strains, but associated with distinct CRISPR arrays with different repeats (gttgagaatcaacaaaggatatgtttaagc and gttttagtttaactatgtgaaatgtaaat) and diverse spacers. Strain *Clostridium novyi* 150557 had only one putative *cas14a* gene and one CRISPR array. Noteworthily, three different types of restriction modification (RM) systems were identified among the 12 strains, located between the two identified *cas14a* genes (Supplementary Figure S1). Furthermore, *cas14a* genes were found to be part of an integrative element splitting the RNA component of RNase P (*rnpB*; Figure 3; Supplementary Figure S1). The detailed analysis of this integrative element revealed a genetic content similar to a phage-inducible chromosomal island (PICI), with the presence of a *Staphylococcus aureus* pathogenicity island (SaPI) repressor homolog, a terminase small subunit, a phage interference redirecting packaging protein (*rppC*) and a minor capsid protein, which are characteristic of PICIs (Penadés and Christie, 2015; Figure 3). This putative PICI was detected in 13 strains (Supplementary Figure S1). In the *Clostridium novyi* NCTC 9693 strain, it was not integrated in *rnpB*, but located 30kb away, upstream from a type I-D CRISPR-Cas system. Furthermore, in *C. botulinum* BoNT/C strain IFR 18/078, this PICI was split by another larger integrative element, which appeared to be plasmid pCN1 from the C-Stockholm strain (Supplementary Figure S1). This putative PICI is the first reported in *C. botulinum*; further investigations are therefore required to confirm its nature and role.

### Identification of Protospacers to Trace Past Encounters of *Clostridium novyi sensu lato* With MGEs

**Figure 4:**
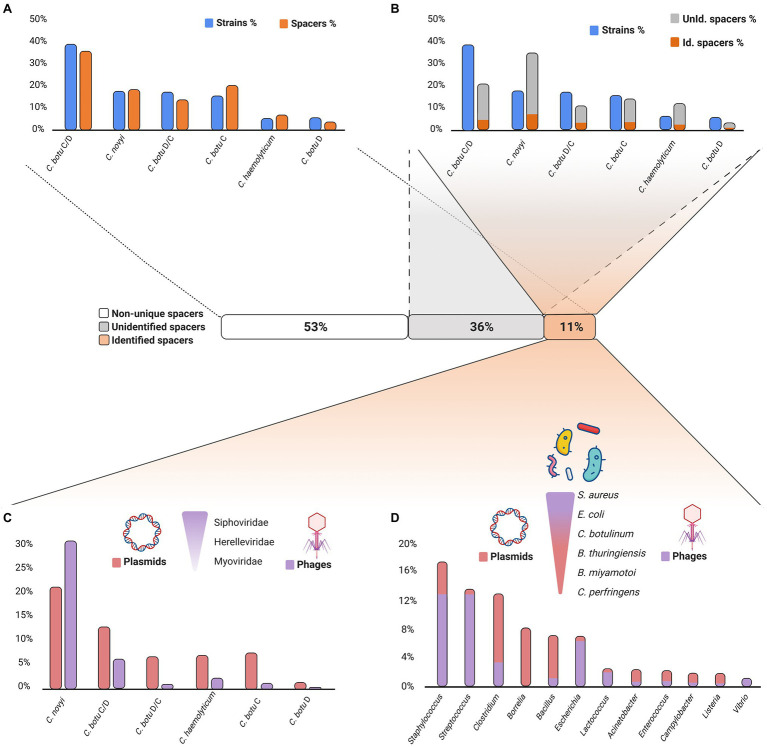
Exploration of *Clostridium novyi sensu lato* spacer content. Created with BioRender.com. **(A)** Distribution of spacers among the analyzed *Clostridium novyi* s.l. strains. Comparison of the distribution of spacers among the analyzed *Clostridium novyi sensu lato* strains. Overall, each species contributed equally to the total number of spacers. Raw data are available in Supplementary Table S1. **(B)** Identified and unidentified unique spacer distribution among the analyzed *Clostridium novyi sensu lato* strains. Comparison of the distribution of identified and unidentified spacers and their species origin based on unique spacer sequences. *Clostridium novyi* spacers are the main contributor to the unique spacer content. The distribution of the identified spacers is not proportional to the number of strains in each species. Raw data are available in Supplementary Tables S3 and S4. **(C)** Distribution of the MGE origins among *Clostridium novyi sensu lato* spacers. Plasmids and phages are equal sources of protospacers. Among the phage protospacer origins, *Siphoviridae*, *Myoviridae*, and *Herelleviridae* predominate. *Clostridium novyi* and *Clostridium botulinum* BoNT C/D are the main analyzed species carrying the protospacers. Raw data are available in Supplementary Tables S6 and S7. **(D)** Targeted MGE host origins among the *Clostridium novyi* s.l. spacers. Exploration of the hosts of the MGEs identified as targets of the CRISPR spacers found in *Clostridium novyi sensu lato*. The MGEs originate mainly from *Clostridium*, *Bacillus*, *Streptococcus*, and *Borrelia* genera. The main species in these genera are *C. botulinum*, *Bacillus thuringiensis*, *Bacillus cereus*, *Staphylococcus aureus Borrelia burgdorferi*, and *Borrelia afzelii*. Raw data are available in Supplementary Tables S8 and S9.

The spacers of *Clostridium novyi sensu lato* CRISPR arrays were also investigated. Spacer arrays indeed represent the memory of the past encounters with MGEs and, by tracing back the spacer origin (the protospacer); they can be used to better understand the mobilome composition/interaction among *Clostridium novyi sensu lato* strains. Spacer sequences were extracted to build a representative database from the 58 strains used in this study (Table 1). We recovered 4,320 spacers, which mostly belonged to *C. botulinum* BoNT C/D (35.8%), *C. botulinum* BoNT/C (20.2%), and *Clostridium novyi* (18.8%), proportional to the number of strains used in our study (Figure 4A; Supplementary Table S1). Shared identical spacers were investigated first (Supplementary Table S2). Interestingly, the distribution of strains based on shared spacers was very similar to the distribution previously obtained using Gegenees (Skarin and Segerman, 2014) or based on SNP phylogeny profiling (Woudstra et al., 2016). For example, 17 *C. botulinum* BoNT C/D strains from lineage Ia shared more than 90% of their spacers (Supplementary Table S2). Analysis of the spacers in strains BKT2873 and BKT75002 (99% whole genome similarity using Gegenees; Skarin and Segerman, 2014) revealed 100% identical spacer content (Supplementary Table S2).

After exploring the shared spacers, we focused on the analysis of the 2,045 unique spacers (47.3%; Supplementary Table S3). Among the unique spacers, the distribution was no longer proportional to the number of strains in each analyzed species, with *Clostridium novyi* spacers now representing up to 34.6% of the unique spacer content and *C. botulinum* BoNT C/D spacers accounting for 20.6% (Supplementary Table S3). This pattern is in concordance with our above observation on spacers widely shared in the *C. botulinum* BoNT C/D lineage Ia, resulting in a lower number of unique spacers, with many of them being present multiple times in genetically related strains.

We then screened the unique spacers *via* a BLAST search using the CRISPRTarget tool (Biswas et al., 2013) to determine their origin (the protospacers) and found 474 spacers matching with at least 80% homology in the GenBank-Phage and Refseq-plasmid databases (Figure 4B; Supplementary Table S4). The remaining 1,571 unidentified spacers (Supplementary Table S5) belong to unknown or uncharacterized MGEs, the so-called “spacer dark matter” (Mcginn and Marraffini, 2019).

A BLAST search on the 474 spacers returned 1,503 protospacer hits in the Refseq-Plasmid and GenBank-Phage databases (Figure 4C; Supplementary Table S6). Most of the identified protospacers originally belonged to spacers from *Clostridium novyi* (51.7%, with 20.9% located in plasmids and 30.8% in phages) and *C. botulinum* BoNT C/D (18.8%, 12.6% located in plasmids, and 6.1% in phages; Supplementary Table S7). The high number of protospacers identified relative to the number of spacer sequences reflects the acquisition of protospacers from homologous genes present in different or related MGEs.

The 1,503 BLAST hits retrieved from the identified protospacers corresponded to 776 unique MGEs (Supplementary Table S8), composed of 379 plasmids (48.8%) and 397 phages (51.2%). Phages were mostly temperate *Siphoviridae* and *Herelleviridae* (among the most common occurring phage species; Supplementary Table S8). The phages ranged in size from 497,513bp for the largest (*Myoviridae* phage G) to 17,637bp for the smallest (*Podoviridae* vB_SauP_phiAGO1.9). MGEs originating from the *Staphylococcus*, *Streptococcus*, *Clostridium*, *Bacillus*, *Escherichia*, and *Borrelia* bacterial genera represented 66.8% of the unique MGEs (Figure 4D; Supplementary Table S9). Interestingly, the bacterial genera varied in the type of MGE contribution (plasmid or phage). *Clostridium*, *Bacillus*, and *Borrelia* mainly contributed plasmids (32.3%), whereas *Staphylococcus*, *Streptococcus*, and *Escherichia* mainly contributed phages (32.5%; Supplementary Table S9). At the species level, *S. aureus*, *Escherichia coli*, *C. botulinum*, *Bacillus thuringiensis*, *Streptococcus thermophilus*, *Clostridium perfringens*, *Lactococcus lactis*, *Staphylococcus epidermidis*, *Acinetobacter baumannii*, *Streptococcus pyogenes*, *Borrelia miyamotoi*, *Clostridium difficile*, *Bacillus cereus*, *Streptococcus agalactiae*, and *Streptococcus mitis* were the main sources of MGEs (Supplementary Table S9B).

### A Limited Number of *C. Botulinum* MGEs Predominate *Clostridium novyi sensu lato* Spacer Sequences

Protospacers identified from MGEs belonging to *C. botulinum* represented 27.6% of all BLAST results, with the highest average BLAST score of 28.8 (Table 2A), therefore suggesting that they are the most recent and frequently encountered ones. Of these MGEs, p1_Cst, p4_BKT015925, p6_Cst, C_Wou-2020a, p1_BKT015925, and p2_BKT015925 represented up to 20.2% of the BLAST results (hereafter called the “restricted mobilome”), showing a roughly similar average BLAST score, suggesting parallel and concomitant dissemination (Table 2B). Although these MGEs belong to different groups of mobile elements (p1_Cst and p1_BKT015925 are pseudolysogenic BoNT phages; Skarin et al., 2011, p4_BKT015925 and p6_Cst have the characteristics of prophage-plasmid hybrids; Pfeifer et al., 2021, CWou-2020a is a circular prophage and p2_BKT015925 is a plasmid; Skarin et al., 2011), they appeared to be genuinely recognized by the CRISPR systems. In a few cases, CRISPR protection was incomplete (e.g., against p4_BKT015925), because the strain had spacers as well as the immunized MGE as part of their genome (Woudstra et al., 2017). This may suggest that these MGEs persist even in the presence of spacers matching with 100% homology, using an unidentified strategy (screening for anti-CRISPR using AcRanker was negative, data not shown; Eitzinger et al., 2020). On the contrary, full CRISPR protection was also observed, e.g., strains that had acquired protospacers against p2_BKT015925 were devoid of the corresponding plasmid (Woudstra et al., 2017).

**Table 2:** The score value represents the percentage of homology between the spacer and its target (Biswas et al., 2013).

Table 2A: Spacer BLAST scores of the main targeted bacterial species.						
Genus	Protospacer species origin	% BLAST results	Average score	St. dev.	Min.	Max.
*Clostridium*	*Clostridium botulinum*	28%	28.76	4.97	21	38
*Staphylococcus*	*Staphylococcus aureus*	11%	21.65	0.80	21	26
*Streptococcus*	*Streptococcus thermophilus*	7%	24.38	1.16	22	26
*Escherichia*	*Escherichia coli*	4%	22.25	0.54	22	24
*Borrelia*	*Borrelia miyamotoi*	2%	22.19	0.68	21	23
*Bacillus*	*Bacillus thuringiensis*	2%	23.84	1.95	21	28
*Clostridium*	*Clostridium perfringens*	2%	23.38	2.88	21	32
*Lactococcus*	*Lactococcus lactis*	2%	23.50	2.15	21	30
*Clostridium*	*Clostridium difficile*	1%	23.00	0.82	22	24
*Staphylococcus*	*Staphylococcus epidermidis*	1%	22.25	0.83	21	23
*Acinetobacter*	*Acinetobacter baumannii*	1%	22.67	0.94	22	24
*Streptococcus*	*Streptococcus pyogenes*	1%	23.27	2.21	21	28
*Bacillus*	*Bacillus cereus*	1%	22.86	1.28	21	25
*Streptococcus*	*Streptococcus agalactiae*	1%	23.87	0.88	22	26
*Streptococcus*	*Streptococcus mitis*	1%	24.36	2.38	22	28
**Table 2B: Spacer BLAST scores of the main targeted *C. botulinum* mobile genetic elements (MGEs).**						
**Mobile genetic element**	**Protospacer species origin**	**% BLAST results**	**Average score**	**St. dev.**	**Min.**	**Max.**
p1_Cst	*Clostridium botulinum*	6%	31.36	4.98	21	38
p4_BKT015925	*Clostridium botulinum*	5%	29.42	4.43	21	38
p6_Cst	*Clostridium botulinum*	4%	30.57	4.69	22	37
CWou-2020a	*Clostridium botulinum*	3%	29.03	5.33	22	38
p1_BKT015925	*Clostridium botulinum*	2%	30.46	3.71	24	36
p2_BKT015925	*Clostridium botulinum*	1%	27.58	3.66	22	34

The higher the score, the better the homology, indicating a putative recent encounter. A: *Clostridium botulinum* is the main target of the spacers with up to 28% of the BLAST results, together with the highest average score. This illustrates the active dissemination of *C. botulinum* mobile genetic elements (MGEs) throughout *Clostridium novyi sensu lato*. B: The most encountered *C. botulinum* MGEs are p1_Cst>p4_BKT015925>p6_Cst, accounting for 15% of the BLAST results. Other *C. botulinum* MGEs (CWou-2020a, p1_BKT015925, and p2_BKT015925) account for 6% of the BLAST results, showing less frequent encounters. The average BLAST scores are roughly equivalent among the *C. botulinum* MGEs, suggesting concomitant dissemination.

In *Clostridium novyi sensu lato*, BoNT-producing strains showed slightly more encounters with the restricted mobilome with 11.1% of BLAST results, whereas non-BoNT-producing strains accounted for 9.1% (Table 3). The localization of spacers targeting the restricted mobilome was mainly chromosomal, but also found in MGEs (Table 3; Supplementary Table S6), indicating that MGEs containing CRISPR arrays may be involved in the protection against these elements. Phage-plasmid hybrids p4_BKT15025 and p6_Cst were the main source of acquired protospacers (8.9%), with 65.9% of BoNT-producing, and 78.6% of non-BoNT-producing strains being immunized. A high number of protospacers was detected in some strains, with up to 16 spacers for *C. haemolyticum* KFSHRC_CH1 targeting the BoNT phages (Table 3; Supplementary Table S6). The protospacers in these MGEs originated mainly from hypothetical genes and intergenic regions (39.4%) with high BLAST scores (Supplementary Table S10). Other protospacers were located in phage-related genes (e.g., capsid, tail, baseplate, terminase, and holin), but also plasmid-partition and replication genes (e.g., ParM, ParB, and replication initiation protein; Supplementary Table S10).

**Table 3:** Spacers targeting the restricted mobilome.

MGE name	MGE type	Localization of spacers	BoNT-producing strains	% of all unique spacers	% of BoNT+−strains containing spacers	Number of spacers/strain
p1_Cst, p1_BKT015925	BoNT phage	97.8% chromosomal, 2.2% MGEs	+	4.2%	84.1%	1–13
			−	3.8%	50.0%	2–16
p4_BKT015925, p6_Cst	Prophage/plasmid hybrid	93.1% chromosomal, 6.9% MGEs	+	5.2%	65.9%	1–11
			−	3.7%	78.6%	2–14
CWou-2020a	Prophage	100% chromosomal	+	1.2%	47.7%	1–5
			−	1.3%	50.0%	1–8
p2_BKT015925	Plasmid	100% chromosomal	+	0.5%	25.0%	1–4
			−	0.3%	28.6%	1

Origin of spacers specifically targeting the restricted mobilome p1_Cst, p1_BKT015925, p4_BKT015925, p6_Cst, CWou-2020a, and p2_BKT015925 were analyzed. BoNT+ strains refer to *C. botulinum* BoNT C, C/D, D, and D/C; BoNT– strains refer to *Clostridium novyi*, *C. haemolyticum*, and *Clostridium* sp. Distribution of spacers with regard to the entire BLAST results, to the proportion of BoNT +/− strains and the number of spacers targeting this restricted mobilome are given. BoNT, botulinum neurotoxin; MGE, mobile genetic element;

BoNT-, *C novyi*, *C. haemolyticum*, and *Clostridium* sp.; BoNT+, others.

### Spacer Sequences in *C. botulinum* Group III MGE CRISPR Arrays

The presence of a type I-B CRISPR-Cas system associated with CRISPR arrays was detected in BoNT phages p1_Cst, p1_1873, p1_BKT015925, p1_BKT2873, p1_16868, and plasmids p2_BKT015925 and p?CLG2/pCN2 (Figure 2; Supplementary Table S11). From the 171 spacers extracted (11.4% of the unique spacers), surprisingly none were shared among any of the different MGEs. Protospacer identification was possible for 28.6%, yielded 133 BLAST hits at an equivalent ratio between phages and plasmids, and belonged mostly to phages from *Escherichia* and *Enterococcus* (41.4%) and to plasmids from *Clostridium* and *Bacillus* (40.6%). *Clostridium botulinum* MGEs (26.3%), with BLAST scores ranging from 22 to 36, predominated, especially p4_BKT015925, p6_Cst, p1_Cst, p1_BKT015925, CWou-2020a, p5_BKT015925, and p2_BKT015925 (18.0%; Supplementary Table S11), in accordance with our previous results (Table 2B). Interestingly, protospacers from p4_BKT015925 were found to have been frequently acquired by CRISPR arrays located on chromosomes or on MGEs, suggesting that *C. botulinum* MGEs have also protected themselves against this potentially invasive element. In addition, BoNT phage acquisition of protospacers from BoNT phages was also detected (p1_16868 vs. p1_BKT015925; p1_BKT015925 vs. p1_Cst; and p1_BKT2873 vs. p1_BKT015925), suggesting that BoNT phages to use the CRISPR system to target other BoNT phages.

### PICI-Related Spacer Content

A type V-U CRISPR was detected in a putative PICI element present in 12 *Clostridium novyi sensu lato* strains from lineage II, containing two distinctive CRISPR arrays (namely CRISPR 1 and CRISPR 2). In the CRISPR arrays, CRISPR 1 contained only 7–9 spacers, and CRISPR 2 had 9–28 spacers. Interestingly, the main spacer diversity was found in *Clostridium novyi*, *C. haemolyticum*, and *Clostridium* sp., which did not share any spacers (Supplementary Table S12). Among the eight *C. botulinum* BoNT/C strains, the PICI sequences were highly conserved (as illustrated in Supplementary Figure S1 for the PICI-Cas14a elements) and had almost exactly the same CRISPR arrays (differences in 1–2 spacers), suggesting that they are highly phylogenetically related. This close relationship was confirmed by comparing whole genome sequences (data not shown), therefore suggesting that all *C. botulinum* BoNT/C strains having a PICI are clonal. When considering the spacers extracted from the two CRISPR arrays, 51.8% were unique. Of the unique spacers, 32.2% gave BLAST results and could be attributed to a protospacer (Supplementary Table S12). Among the identified protospacers, 18 with the best BLAST score (25–36) belonged to the above-identified restricted mobilome (Table 2B). Interestingly, one spacer located in the *C. botulinum* BoNT/C Stockholm strain in the PICI CRISPR array one targets its own BoNT phage (BLAST score 30) as well as plasmid pCLG2/pCN2 (BLAST score 28; Supplementary Table S12). Other identified spacers were mainly acquired by the CRISPR arrays from PICIs in *Clostridium novyi* (7), *C. haemolyticum* (5), and *Clostridium* sp. strains (5), which did not possess these MGEs as part of their genomes (Supplementary Table S12).

## Discussion

### *Clostridium novyi sensu lato* Species Harbor a Range of CRISPR-Cas Systems

The first objective of our study was to investigate the diversity and composition of *Clostridium novyi sensu lato* CRISPR-Cas systems, which had not been fully explored yet (Woudstra et al., 2016; Negahdaripour et al., 2017). We detected a high prevalence of CRISPR-Cas systems (complete or partial) throughout the 58 strains included in our study, with only three strains lacking CRISPR-Cas systems (*C. botulinum* It1, *Clostridium novyi* BKT29909, and *Clostridium novyi* 4540, all lineage IV). Accordingly, a CRISPR prevalence of 80% was detected in *C. botulinum* strains, in a study including only eight *Clostridium novyi sensu lato* genomes (Negahdaripour et al., 2017). This prevalence is much higher than the rate of 46% reported for bacteria (Grissa et al., 2007) or other clostridia species such as *C. perfringens*, which showed a CRISPR prevalence of 53.15% (Long et al., 2019). The location of some CRISPR-Cas systems on MGEs may play a role in this high prevalence. Some strains, in particular from lineage Ia, only have CRISPR-Cas systems in MGEs and none in their chromosome.

In our study, the number of CRISPR loci varied between one and three per strain, which seems to be common in pathogenic bacteria (Cady et al., 2011; Shariat et al., 2013; Yin et al., 2013; Karah et al., 2015), and belonged to class 1 and 2. Class 1 CRISPR-Cas systems (types I-B, I-D, III-B, and III-D) were identified in the *Clostridium novyi sensu lato* strains investigated in our study, similar to previous studies that have shown that the type I CRISPR-Cas system is the most common type found in clostridia (Makarova et al., 2020) and that type III-B, followed by type I-B, is the most commonly identified types in *C. botulinum* groups I and II (Negahdaripour et al., 2017). It is noteworthy that only one type of CRISPR-Cas system (I-B) was detected in lineages Ia, III, and IV, but several types were detected in lineages Ib and II. Previous studies have shown that the number of MGEs is higher in *C. botulinum* BoNT C/D (lineage Ia) than in BoNT D/C (lineage Ib) containing several different CRISPR types (Woudstra et al., 2016, 2017). In *B. cereus*, strains with active CRISPR-Cas systems have fewer MGEs than strains with partial CRISPR-Cas systems or without any CRISPR-Cas systems (Zheng et al., 2020). Likewise, CRISPR-Cas systems may play a barrier role against horizontal gene transfer by preventing the acquisition of MGEs, resulting in a lower occurrence of plasmids and phages in strains from lineage Ib, and the opposite in lineage Ia strains.

Type I-B CRISPR loci (either complete or incomplete) were the most common CRISPR type detected in *Clostridium novyi sensu lato*. Type I-B seems to be typical of clostridia. It has indeed been detected in *C. perfringens* (Long et al., 2019) as well as in *C. chauvoei* (Thomas et al., 2017), *C. tetani* (Cohen et al., 2017), and in all *C. difficile* genomes (Hargreaves et al., 2014; Andersen et al., 2016). Here, several type I-B CRISPR arrays were also located on MGEs such as the BoNT phages and the plasmid p2_BKT015925. The localization in prophages has also been reported in *C. difficile* (Hargreaves et al., 2014; Boudry et al., 2015). Prophage localization of CRISPR arrays may play a role in preventing infection by related competing phages (Sorek et al., 2008). It may also play a role in the mobilome dynamics in *Clostridium novyi sensu lato*, in particular in lineage Ia for which the localization of a partial type I-B CRISPR in the pseudolysogenic BoNT phage was preponderant, whereas no CRISPR locus was detected chromosomally. Noteworthily, type I-B CRISPR loci located on BoNT phages in BoNT C/D strains was incomplete, lacking the adaptation modules (Cas1, Cas2, and Cas4). In a previous study, putative type I-related operons, derivatives of subtype I-F or subtype I-B encoding interference *cas* genes, but not associated with *cas1*, *cas2*, or *cas3* genes, have been detected (Makarova et al., 2015). They are located either on plasmids or associated with transposon-related genes (Makarova et al., 2015). Here, they were located in plasmids and in the BoNT phages sometimes associated with transposases. The functionality of these CRISPRs was not experimentally investigated in our study, but the presence of various spacers in CRISPR arrays suggests they are active. The absence of the adaptation module (Cas1, Cas2, and Cas4) would only be detrimental to the acquisition of new spacers, and it would not prevent the effector complex from performing its function, except in the absence of Cas3. Without Cas3, the effector complex is not able to cleave its target – but only bind –, which may result in transcription interference (CRISPRi; Hawkins et al., 2015). The presence of complete CRISPR-IB systems in the chromosome for some strains may also compensate for the missing Cas proteins, allowing these incomplete systems to be fully functional (e.g., in the *C. botulinum* group III Stockholm strain). Tranposases showing similarities with TnpB and Cas14a and interrupting the CRISPR loci were detected in some strains. This is intriguing because Cas14a has been shown to be capable of both transposase- and CRISPR-related activities (Shmakov et al., 2017). Further investigations are required to determine the exact role of these transposases.

There may be other biological significance for these incomplete CRISPR-Cas systems located on MGEs, as roles other than immunological protection have been revealed for CRISPR-Cas systems, such as signal transduction and gene regulation (Bikard et al., 2012). Considering that some CRISPR locus located on MGEs can lose their endonuclease, the cascade complex would only be able to bind its target, therefore promoting steric transcriptional inhibition (Zhang et al., 2021). Further experimental investigations are required to better understand the role and mechanisms of these incomplete type I-B systems located in MGEs in the biology of *Clostridium novyi sensu lato*.

In addition to type I-B, other CRISPR-Cas system subtypes were found in *Clostridium novyi sensu lato* strains, except *C. botulinum* BoNT C/D (lineage Ia) and strains from lineages III and IV. Type I-D was found in *C. botulinum* BoNT/D (33%) and D/C (90%) and in *Clostridium novyi* (20%; lineages Ib and II, Figure 1). Considering the low number of available BoNT D/C genomes (currently 10 genomes in public databases), it is difficult to draw conclusions and establish generalities. However, based on the analysis of these genomes, a complete type I-D system appears to be preponderant mostly in *C. botulinum* BoNT D/C strains (lineage Ib). This CRISPR-Cas system is a hybrid of type I and type III systems (Lin et al., 2020; Mcbride et al., 2020), most often reported in cyanobacteria and archaea (Lin et al., 2020). So far, little is known about this subtype and its biological properties (Lin et al., 2020).

Type II-C was the second-most common CRISPR type found in *C. botulinum* BoNT D/C strains (60%) in lineage Ib. This class II CRISPR system is detected in diverse bacterial species in various environments, especially in pathogenic and commensal bacteria (Mir et al., 2018). A possible role in virulence for type II-C CRISPR-Cas systems has been suggested (Mir et al., 2018), but there is no experimental evidence for such function in *Clostridium novyi sensu lato*. Because *C. botulinum* genome engineering has been successful using CRISPR type II-B Cas9 (Mertaoja et al., 2021), type II-C can also be explored as a native alternative for genome modification using a mini-plasmid strategy (Pyne et al., 2016). This strategy may be advantageous, as modification of clostridia is cumbersome.

Finally, CRISPR type III was also found in *Clostridium novyi sensu lato* and is represented in numerous archaea but is less frequent in bacteria (Koonin et al., 2017), except in *C. botulinum* groups I and II where type III-B has been reported to be preponderant (Negahdaripour et al., 2017). Type III systems degrade both DNA and RNA (Pyenson and Marraffini, 2017; Tamulaitis et al., 2017; Terns, 2018). Here in our study, type III-B and III-D systems were detected in four strains (lineages Ib and II). They lacked *cas1* and *cas2* genes, which seems to be common in type III-B, C, and D systems (Makarova et al., 2015), especially in genomes also containing type I CRISPR-Cas loci. Due to their plasticity in crRNA selection, type III-B interference machinery can use type I spacers to counter infection from bacteriophages that have escaped the type I defenses through PAM mutations (Silas et al., 2017). Here, the four genomes in which these CRISPR systems were detected also had type I-B and type I-D systems, which suggest such a mechanism for these strains.

Lastly, two putative type V-U CRISPR *cas14a* genes encoding homologous proteins associated with two distinct CRISPR arrays were detected in 81.25% of *Clostridium novyi sensu lato* strains belonging to genomic lineage II, and not in other lineages. Type V-U CRISPR targets single-stranded DNA (ssDNA) sequences without requiring restrictive signal sequences (Khan et al., 2019). The ability of Cas14a to specifically target ssDNA suggests a role in defense against ssDNA viruses or MGEs that propagate through ssDNA intermediates, which may be an advantage in some ecosystems containing ssDNA viruses (Harrington et al., 2018), such as soils (Kim et al., 2008), water (Angly et al., 2006; Rosario et al., 2009), or human and farm-animal feces (Sikorski et al., 2013). Here no ssDNA viruses were identified among the spacers located in the two CRISPR arrays from the putative type V-U CRISPR Cas14a systems with a BLAST match (Supplementary Table S12). This absence does not exclude the possibility of finding such ssDNA targets among the spacers with no BLAST matches, but there may be other roles for these putative Cas14a systems. For example, the localization of CRISPR-arrays close to the Cas14a genes may suggest that Cas14a plays a role in the spacer acquisition step.

The presence of this putative type V-U CRISPR only in strains from genomic lineage II suggests a specific mechanism of horizontal transfer for this lineage. Moreover, it was included in a putative PICI. PICIs are a recently discovered family of pathogenicity islands that contribute to horizontal gene transfer, host adaptation, and virulence in Gram-positive cocci, but are also widespread among Gram-negative bacteria (Fillol-Salom et al., 2018). These highly evolved molecular parasites can hijack phage-packaging systems, thus allowing its transfer to other bacterial cells (Crestani et al., 2020). In doing so, PICIs protect the host resident population against specific phages. Although the insertion region was present in all *Clostridium novyi sensu lato* genomic lineages (data not shown), only lineage II possessed the putative PICI-Cas14a element, which suggests a narrow host-range dissemination. Furthermore, the presence of diverse spacer sequences in the PICI CRISPR arrays indicates that the system actively acquires protospacers from invading MGEs. The high protospacer identification BLAST score suggests recent acquisition, especially in the identified restricted mobilome. Interestingly, all *C. botulinum* BoNT/C strains having a PICI were clonal with highly conserved PICI sequences; despite being isolated from different countries and years (1946 in Sweden for strain Stockholm and 1970 in United Kingdom for strain Colworth BL165 for example). This might suggest a high stability of these genomes, including the PICI sequences but more genomic data of *C. botulinum* BoNT/C lineage II strains are required to better explore and understand this result.

Noteworthily, an RM system was associated with the putative *cas14a* genes that may add another layer of defense for the PICI host. Transfer of genes encoding non-essential or accessory functions by PICIs has been previously reported (Penadés and Christie, 2015), but to our knowledge, this is the first time that CRISPR and RM systems have been encountered in a PICI. Further investigations are required to study this putative PICI element and its role in *Clostridium novyi sensu lato* lineage II ecology in more detail.

### Protospacer Origins Reveal a Handful of MGEs That Are Widespread Among *Clostridium novyi sensu lato* Species

The second objective of our study was to investigate the spacers found in the CRISPR arrays of *Clostridium novyi sensu lato*. Their analysis revealed a high correlation with strain distribution previously obtained using other tools, in particular the distribution of genetic lineages (Skarin and Segerman, 2014; Woudstra et al., 2016). Strains previously identified as being closely related by genomic comparison indeed harbor the same or almost the same spacers (Skarin and Segerman, 2014; Woudstra et al., 2016). Genotyping spacers thus holds promise for identifying common ancestors among strains, which would allow strain tracking with high resolution and accuracy or highlight potential relatedness between isolates. This approach has been used for several pathogens such as *Campylobacter* (Kovanen et al., 2014), *Salmonella* (Xie et al., 2017), or *C. difficile* (Andersen et al., 2016).

A large proportion of the identified unique spacers originated from *Clostridium novyi* and *C. botulinum* BoNT C/D strains. However, the current number of available genomes of *Clostridium novyi sensu lato* is quite low (58) and the lineages, BoNT types and species are unequally represented. Detection of most of the unique spacers in *Clostridium novyi* and *C. botulinum* BoNT C/D genomes may thus be linked to the high number of strains or high diversity observed in both species. Among the few previously analyzed genomes (Skarin and Segerman, 2014), *Clostridium novyi* and *C. botulinum* BoNT C/D were distributed throughout three different lineages, whereas *C. botulinum* BoNT/D strains were classified into two lineages and *C. botulinum* type C, type D/C and *C. haemolyticum* in only one single lineage. This may explain why the detection of unique spacers was higher in *Clostridium novyi* and *C. botulinum* BoNT C/D than in the other genomes. However, more genomes are needed to confirm this observation. The high diversity of spacers detected in *Clostridium novyi* genomes despite the low number of available genomes (10) may also be linked to the high diversity of strains included in our study. They were isolated between 1920 and 2015, from five different countries and five different animal species.

Among the spacers whose origin was identified, plasmids and phages were found in equivalent proportions (respectively 51.2 and 48.8% of all MGEs), as already previously reported in *C. botulinum* group I, II, and III (Negahdaripour et al., 2017). This distribution is different from what is commonly reported in prokaryotes, where 85% of spacers usually map to phages (Mcginn and Marraffini, 2019), including in other clostridia species such as *C. perfringens* (Long et al., 2019). In addition, some MGEs of *Clostridium novyi sensu lato* contain proteins characteristic of phages as well as plasmids (e.g., p1_BKT015925, p1_Cst, p4_BKT015925, and p6_Cst), resulting in misleading annotations in public databases, which may have artificially inflated the proportion of spacers from plasmids.

The exploration of CRISPR spacer sequences allowed the identification of their protospacers and thus past encounters of *Clostridium novyi sensu lato* with MGEs. The hosts from which the targeted MGEs originate provided partial information on the environmental microbiome composition in which *Clostridium novyi sensu lato* thrived and on the bacterial species with which MGEs are commonly exchanged. The main bacterial species from which the MGEs targeting *Clostridium novyi sensu lato* originated included *S. aureus*, *E. coli*, *C. botulinum*, *B. thuringiensis*, *B. miyamotoi*, *C. perfringens*, *S. epidermidis*, and *S. thermophilus* (38.1% of the total BLAST results). BLAST scores were lower for *Borrelia* than for *Clostridium* and *Bacillus* reflecting putative distant matches. *Clostridium botulinum* was the primary source of the main MGEs detected in this study, with the best BLAST scores, reflecting active dissemination of their MGEs throughout *Clostridium novyi sensu lato*.

The analysis of protospacers also provides information on the ability of MGEs to disseminate successfully within a bacterial species. Of the 776 unique MGEs identified from the identified protospacers, only six of them represented up to 20.2% of the total BLAST results with the best BLAST scores, namely, phage-plasmid hybrids (p4_BKT015925, p6_Cst), BoNT pseudolysogenic phages (p1_Cst, p1_BKT015925), prophages (C_Wou-2020a), and a plasmid (p2_BKT015925) originating from *C. botulinum*. Considering the large mobilome of *Clostridium novyi sensu lato* (Skarin and Segerman, 2014), it may be surprising that only a handful of MGEs are responsible for such large protospacer acquisition. This may imply that these elements are highly transferable throughout *Clostridium novyi sensu lato*. Although spacers targeting p1_Cst, p1_BKT015925 were detected in 84.1% of *C. botulinum* group III strains and 50% of non-toxic *Clostridium novyi sensu lato* strains (Table 3), no spacer targeting the BoNT phage from strains 1873, BKT2873, or 3859 was detected, suggesting no encounters between available sequenced strains and these MGEs. Half of the non-toxic strains did not have any spacers targeting p1_Cst, p1_BKT015925, and neither did six out of the seven lineage IV strains, suggesting no encounter between these strains and p1_Cst and p1_BKT015925, perhaps due to their presence in different ecological niches. This also suggests that these non-toxic strains do not have CRISPR-Cas systems trained against the BoNT phage from the genomes currently available in the database and may therefore potentially be infected by these MGEs. Movements of plasmids and toxin genes across lineage boundaries have been reported in *Clostridium novyi sensu lato* strains (Skarin and Segerman, 2014).

### Limited Acquisition of Mobilome Protospacers Results in Variable Protection Outcomes

Regarding spacers from CRISPR arrays located either in the chromosome or in MGEs, two main scenarios were encountered: (1) the genome containing the spacer(s) is devoid of the MGE carrying the protospacer; (2) the genome containing the spacer(s) also contains the protospacer MGE. A good example of the first situation is the plasmid p2_BKT015925. Protospacers from p2_BKT015925 were indeed only present in strains devoid of the plasmid. In that case, it seems that the CRISPR system efficiently prevents the acquisition of p2_BKT015925 and plays its expected immunological role, demonstrating the active part CRISPR-Cas systems play to regulate the presence or absence of a specific MGE in *Clostridium novyi sensu lato* strains. This was also the case for prophage CWou-2020a, which was only detected in strains that had not acquired CWou-2020a protospacers. Interestingly, several *C. botulinum* BoNT C/D strains had a CWou-2020a homologous prophage (NZ_AESB01000027) present in their genome, but only strains that had not acquired CWou-2020a protospacers, with incomplete type I-B CRISPR system located on MGEs. The absence of spacers targeting CWou-2020a in strains harboring NZ_AESB01000027 may be related to the mutual exclusion of both prophages, either by integrating in the same location or by encoding multiple infection exclusion proteins (Bondy-Denomy et al., 2016), therefore avoiding the need for a CRISPR system defending specifically against CWou-2020a.

The second situation was observed for p1_BKT015925, p4_BKT015925 as well as p6_BKT015925 and may be considered a paradox, because both targets and the corresponding spacers were detected at the same time. Induction of autoimmunity and elimination of the targeted MGE should be expected from such situations. Previous studies have reported the detection of self-targeting spacers (targeting host chromosomal DNA) in many bacterial species (Wimmer and Beisel, 2020). The exact impact of self-targeting is unknown and may be minor. Moreover, some studies have reported examples in which a self-targeting spacer can be tolerated, for example through the activation of effective DNA repair or through mutations or even deletions of targets from the genome (Wimmer and Beisel, 2020). Alternatively, these self-targeting spacers may have biological roles other than immunity such as evolution or RNA degradation (Wimmer and Beisel, 2020). For example, three spacers from *C. botulinum* BoNT C/D strains localized in the chromosome target p1_BKT015925 and had this BoNT phage within their genome. Interestingly, these spacers specifically target variable regions of p1_BKT015925, suggesting that this may be part of a mechanism involved in the prevention of superinfection by several related BoNT phages at the same time. The existence of such a mechanism to prevent superinfection by multiple BoNT phages is also supported by the detection of protospacers from BoNT phages and located on other BoNT phages. For example, spacer 16868_D_90 (present in strains *C. botulinum* BoNT D 16868, BoNT D/C 1274, 1275, and 1276) located on the BoNT D and D/C phages target BoNT C/D phage p1_BKT015925 on the CRISPR Cas7 gene (with four mismatches). The detection of spacers targeting p4_BKT015925 found on BoNT phage p1_BKT015925 and plasmid p2_BKT015925 suggests this kind of competition between MGEs (Supplementary Tables S10 and S11).

## Conclusion

In the present study, the exploration of the CRISPR-Cas systems in the *Clostridium novyi sensu lato* genospecies found six different CRISPR-Cas system types (mainly I-B) belonging to class 1 and 2 CRISPR loci, protecting against up to 776 MGEs, but only a handful of them being widespread. Although the CRISPR mechanism and CRISPR-MGE interaction have been largely investigated, there is much less information on CRISPR systems used by MGEs targeting other MGEs, whose functions remain largely uncharacterized (Faure et al., 2019). This study highlighted that CRISPR-Cas systems are numerous in *Clostridium novyi sensu lato* strains, and may be present either in the bacterial chromosome or in MGEs. The components carrying CRISPR-Cas systems seemed to have been recruited as anti-MGE systems and for inter-MGE conflicts, to protect mainly against a restricted number of MGEs. The role and function of these CRISPR-Cas systems in the bacterial life cycle and more generally in pathogenesis, in particular when located on MGEs, now need to be explored. Furthermore, studying this restricted mobilome may prove very useful for biocontrol strategies against the pathogenic *Clostridium novyi sensu lato* species.

## Data Availability Statement

The datasets presented in this study can be found in online repositories. The names of the repository/repositories and accession number(s) can be found in the article/Supplementary Material.

## Author Contributions

CW designed and coordinated the study. CM is the recipient of a grant that funded this study. TG, CW, and MD performed the bioinformatics analysis. TG, CM, MD, MC, and CW participated in data analysis and interpretation of the results. CM, MC, and CW supervised the study. CW, TG, and CM wrote the manuscript. All authors contributed to the article and approved the submitted version.

## Funding

This study was supported by the “Institut Carnot AgriFood Transition” (TRACK’SPORE project), the French National Research Agency, and the Bretagne Regional Council of Région Bretagne. TG is the recipient of a PhD grant from ANSES and “Région Bretagne.”

## Conflict of Interest

The authors declare that the research was conducted in the absence of any commercial or financial relationships that could be construed as a potential conflict of interest.

## Publisher’s Note

All claims expressed in this article are solely those of the authors and do not necessarily represent those of their affiliated organizations, or those of the publisher, the editors and the reviewers. Any product that may be evaluated in this article, or claim that may be made by its manufacturer, is not guaranteed or endorsed by the publisher.

## Footnotes

^1^ http://acranker.pythonanywhere.com/

## Supplementary Material

Supplementary Table S1 *Clostridium novyi sensu lato* total spacer content.

Supplementary Table S2 *Clostridium novyi sensu lato* duplicated spacer content.

Supplementary Table S3 *Clostridium novyi sensu lato* unique spacer content.

Supplementary Table S4 *Clostridium novyi sensu lato* unique identified spacer origin.

Supplementary Table S5 *Clostridium novyi sensu lato* unique unidentified spacer origin.

Supplementary Table S6 BLAST results on *Clostridium novyi sensu lato* unique spacer origins.

Supplementary Table S7 Proportion of MGEs among the BLAST results on the recognized spacers.

Supplementary Table S8 Composition of MGE host genus origins.

Supplementary Table S10 Main targeted mobile genetic element (MGE) genes.

Supplementary Table S11 Identification of mobile genetic element (MGE) spacer (protospacer) origin.

Supplementary Table S12 Identification of phage-inducible chromosomal island (PICI) spacer (protospacer) origin.
