# Supplementary figures and images for: Exploration of the Diversity of Clustered Regularly Interspaced Short Palindromic Repeats-Cas Systems in Clostridium novyi sensu lato
Source: Front Microbiol. 2021 Sep 13;12:711413. doi: 10.3389/fmicb.2021.711413 (PMC8473940; doi:10.3389/fmicb.2021.711413)

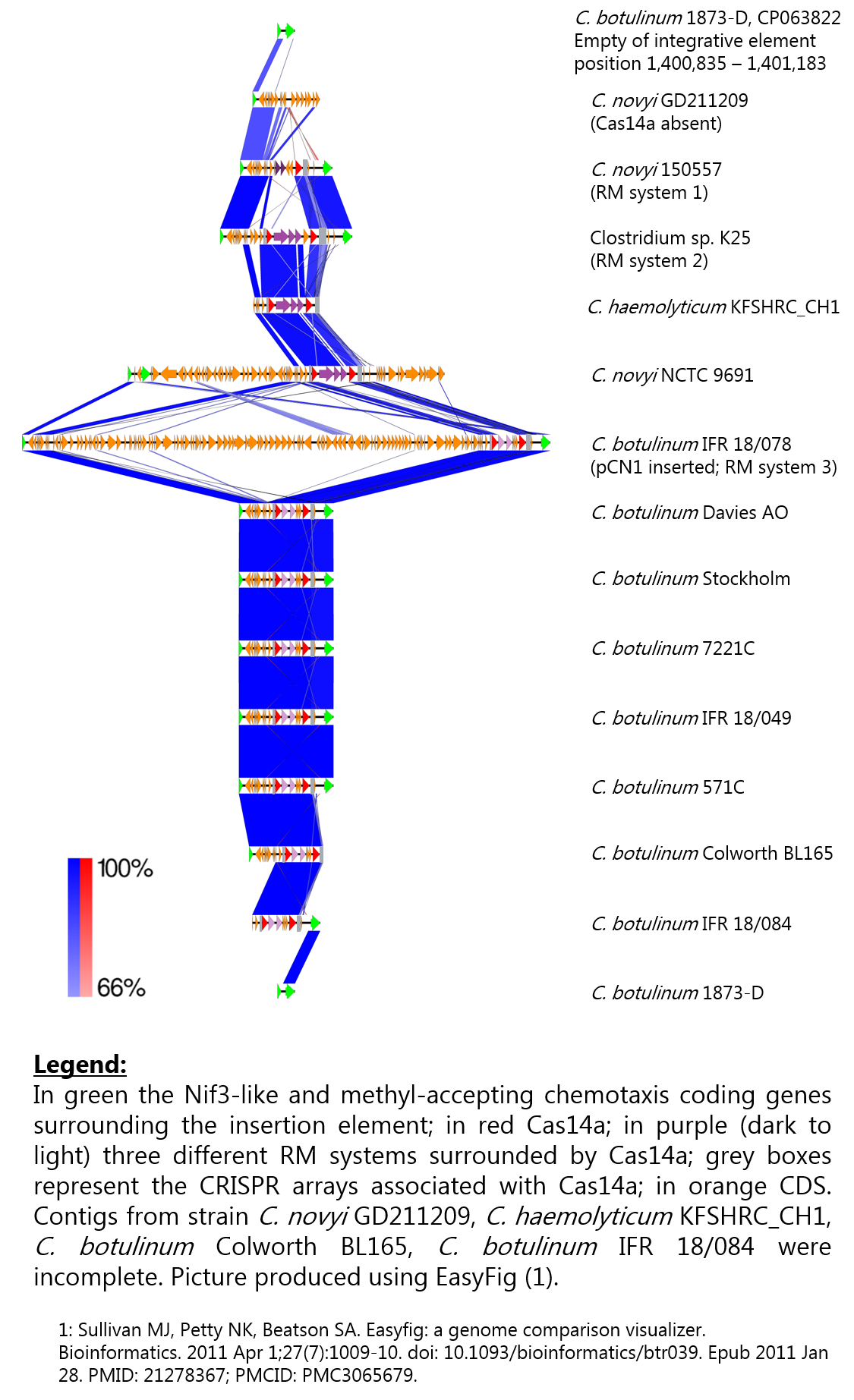

Supplement: Supplementary file 2 [file Image_1.TIF]
